# Compliance considerations in the geo-enrichment of an EHR data warehouse with social and environmental determinants of health

**DOI:** 10.1017/cts.2024.521

**Published:** 2024-05-02

**Authors:** Maryam Abdallah, Neil Bahroos, Praveen Angyan, Beau MacDonald, Camilla Catignas, Daniella Garofalo, Amy Chuang, Hakob Abajian, John Wilson

**Affiliations:** 1 Keck Medicine of USC, University of Southern California, Los Angeles, CA, USA; 2 Southern California Clinical and Translational Science Institute, Los Angeles, CA, USA; 3 Division of Bioinformatics, Department of Preventive Medicine, Keck School of Medicine, University of Southern California, Los Angeles, CA, USA; 4 Spatial Sciences Institute, University of Southern California, Los Angeles, CA, USA

**Keywords:** Compliance, data warehouse, electronic health records, informatics, social determinants of health

## Abstract

Social and environmental determinants of health (SEDoH) are crucial for achieving a holistic understanding of patient health. In fact, geographic factors may have more influence on health outcomes than patients’ genetics. Integrating SEDoH into the electronic health record (EHR), however, poses notable technical and compliance-related challenges. We evaluated barriers to the integration of SEDoH in the EHR and developed a privacy-preserving strategy to mitigate risk of protected health information exposure. Using coded identifiers for patient addresses, the strategy evaluates an alternative approach to ensure efficient, secure geocoding of data while preserving privacy throughout the data enrichment processes from numerous SEDoH data sources.

## Introduction

Electronic health records (EHRs) are inherently limited in providing valuable information for social and environmental determinants of health (SEDoH), though such data are critical for comprehensive patient history and precision medicine initiatives. An individual’s zip code may be linked more to influencing health outcomes or issues than their genetics [1]. Improving our ability to capture SEDoH can bridge the gap in health disparities and improve outcomes for marginalized populations [2,3]. Although some healthcare organizations have integrated patient-reported social determinants forms in their EHRs, data are often sparse [4]. While publicly accessible neighborhood-level SEDoH data exist, seamlessly integrating this information in the patient electronic health record (EHR) is complex and presents compliance-related challenges.

Collecting SEDoH data begins with geocoding, or translating, an address or Census tract to its latitudinal and longitudinal coordinates [5]. Geocoded addresses are then geo-enriched with SEDoH data retrievable through extensive and publicly available datasets, but accurately linking the variables to patient data requires disclosing individual geographic identifiers. Once addresses are geocoded, they can be linked to corresponding neighborhood and community-level SEDoH variables derived from a multitude of datasets. Datasets are available via publicly hosted files, public application programming interfaces (APIs), and commercial APIs that are behind a paywall. These datasets often utilize geolocations defined by the US Census Bureau to report on various SEDoH [4], and some initiatives have combined multiple data sources to create composite SEDoH indices [6]. Geographic identifiers beyond the first three digits of some zip codes are considered protected health information (PHI). Use and disclosure of PHI beyond the scope of providing patient care are restricted based on the Health Insurance Portability and Accountability Act of 1996 (HIPAA) Privacy Rule [7].

Geographic information system (GIS) software can geocode and enhance addresses with geospatial data [1]. The Social and Environmental Determinants Address Enhancement (SEnDAE) toolkit [8] employs an innovative strategy whereby an intermediate server separates the requesting health provider organization’s (HPO) IP address when transmitting deidentified patient addresses to a cloud-based geocoding service [9]. While this significantly reduces the risk of accidental PHI disclosure, further safeguards can be put in place by carrying out in-house geocoding within a self-contained GIS application. Conservative arguments trust that a self-contained approach may be the only HIPAA-compliant method to protect PHI during external data transfer [10]. This paper explores these compliance challenges and offers recommendations for integrating SEDoH with EHR data while minimizing risk.

## Materials and methods

To retrieve SEDoH variables for any individual patient, their home address must be translated, or geocoded, from its standard format into the specific latitudinal and longitudinal coordinates. The geocoded location can then be linked, or geo-enriched, with its corresponding social and environmental data points. This process can be completed either by sending location data to a web service or purchasing a local geocode database. The former option simplifies the process of geocoding, as there is no server set up, installation or maintenance required. Several such services exist, including a free service provided by the US Census Bureau [4]. However, utilizing web services involves risks associated with the disclosure of PHI to a remote server external to one’s institution. The alternate option is to instantiate a local geocode database and service, which can be purchased from several companies. While this requires the additional steps of setup and maintenance of the server, and keeping the software up to date, it eliminates the need for external disclosure of PHI.

**Figure 1.**
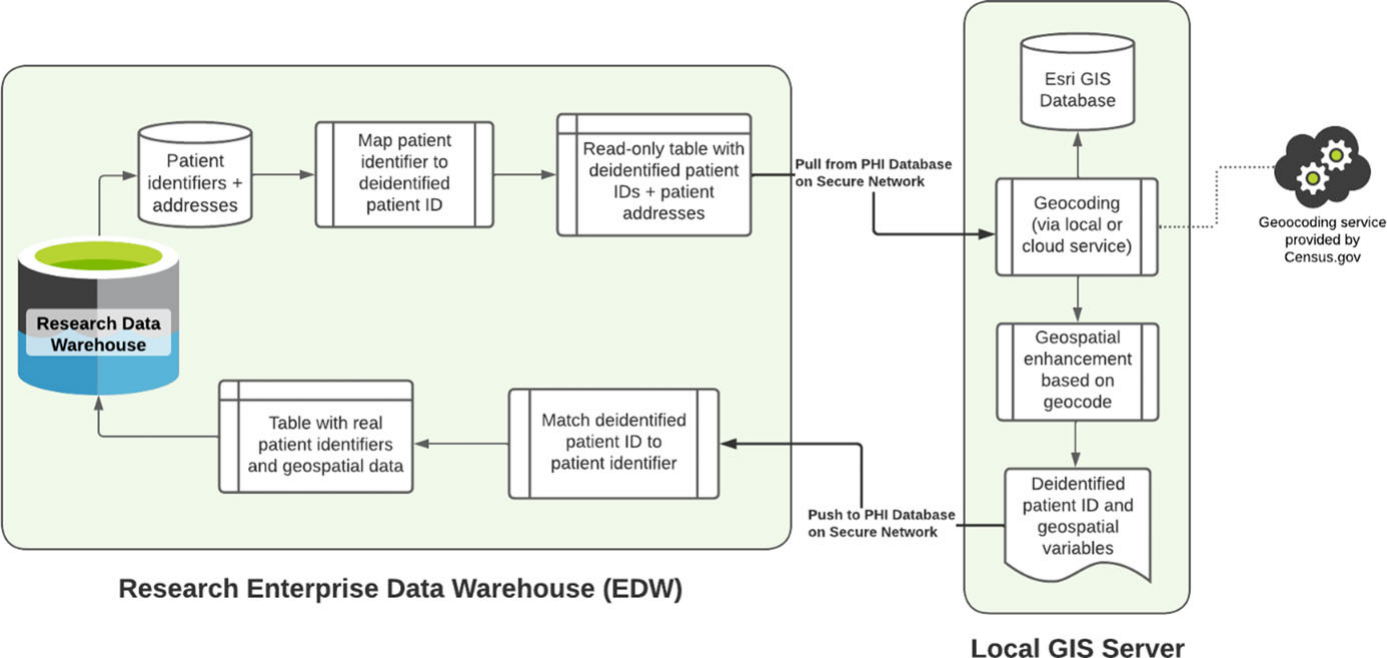
Diagram of workflow utilized for geocoding and geo-enrichment.

The current project purchased Esri’s ArcGIS Pro 3.X with the Business Analyst Extension. The software was installed locally on a secure server created specifically for geocoding and geo-enrichment purposes. We designed a workflow, illustrated in Figure 1, to ensure the local GIS enhancement server contained only the minimum-necessary PHI required for geocoding and geo-enrichment. First, a randomized, deidentified ID is assigned to each patient, yielding a code key that is stored within our HIPAA-compliant Research Enterprise Data Warehouse (EDW). Second, deidentified patient IDs and their corresponding geographical addresses are loaded onto the local GIS server. Third, addresses are geocoded and subsequently assigned a randomized, deidentified address ID, yielding a code key that is stored within the local GIS server. Finally, deidentified address IDs are linked to corresponding Census Tract IDs and exported from the server. Census Tracts do not contain fixed individual geographic identifiers and are considered less specific geographic subdivisions than latitude and longitude, or even Census Block groups [5], further minimizing PHI risk.

## Results

All patients (n = 554,562) within the university’s EHR who opted in to participating in research and had valid addresses were included in this project. Full addresses and deidentified patient IDs were loaded onto the local GIS server. All current and previous patient addresses were included in the data, such that some patient IDs corresponded to multiple addresses. All addresses were geocoded and assigned a randomized address ID.

Datasets were selected from six data sources (Table 1). These sources were determined to contain valuable social and environmental variables and had the necessary geographic identifiers needed for linkage. All datasets were downloaded and stored locally, allowing geo-enrichment efforts to be completed on the local GIS server. Five of the six datasets were directly downloaded from the source and stored on the local server, which took about 10 seconds per dataset. The remaining dataset, the US Census American Community Survey (ACS), was only accessible through API calls and could not be directly downloaded. The ACS API was called with broad arguments to collect data for all addresses across the entire United States. ACS data were downloaded once a month, although it can be refreshed at any frequency as feasible for an institution. A free API key was registered, which the US Census requires for IP addresses that exceed 500 daily queries. Loading all ACS data on the local server via API calls took 8.28 minutes. While this meant that live data were not being obtained through the API, it allowed us to maintain the same level of privacy as downloadable, locally stored datasets.

**Table 1.** Sample data sources included in geo-enrichment of patient addresses

Data Source	Access methodology	Links
US Census American Community Survey, 5-year detailed tables (B), subject tables (S), and data profiles (DP)	API	https://www.census.gov/data/developers/data-sets/acs-5 year.2018.html
Office of Environmental Health Hazard Assessment (OEHHA), on behalf of the California Environmental Protection Agency (CalEPA)	File	https://oehha.ca.gov/calenviroscreen/report/calenviroscreen-40
Centers for Disease Control and Prevention. Agency for Toxic Substances and Disease Registry	File	https://www.atsdr.cdc.gov/placeandhealth/svi/
NASA Air Quality files	File	https://sedac.ciesin.columbia.edu/data/collection/aqdh/sets/browse
Southern California Environmental Health Sciences Center	File	https://scehsc.usc.edu/
US Department of Agriculture, Economic Research Service	File	https://www.ers.usda.gov/data-products/food-access-research-atlas/

Once all addresses were geo-enriched with corresponding variables from all datasets, deidentified patient IDs and linked geospatial data were exported from the local GIS server and loaded back into the Research EDW. Patient IDs were reidentified using the code key maintained within the Research EDW, and the newly geo-enriched patient data were integrated with our existing EHR data warehouse. The data were integrated with our Observational Medical Outcomes Partnership (OMOP) Common Data Model (CDM) [11] by modeling SEDoH data on our local concepts for extending the OMOP CDM and creating measurement tables. We also converted the SEDoH data from OMOP into observations in our local instance of i2b2 [12], a self-service cohort discovery tool, using the SEnDAE ontology extension framework [8]. These efforts allowed for geo-enriched EHR data to be readily available for researchers and clinicians to query and extract. The toolkit and OMOP CDM are publicly available at https://github.com/scctsi/gis-toolkit.

## Discussion

This project was successful in geocoding and geo-enriching an EHR data warehouse in a secure, compliant manner. Utilizing the Esri database provided a minimal-cost solution to support this project. While local installation of the Esri database prevented external PHI transfer, there are limitations to this method. The setup, installation, and maintenance of such a server can be a burden to organizations. Due to the time-consuming nature of the in-house geocoding process and quality validation, our organization currently completes geocoding and geo-enrichment on an ad hoc basis as a consultation service for research projects and once every two years for our entire patient population.

To streamline the geocoding process, we plan to transition to a secure process that utilizes APIs to an external service for geocoding. This has been reviewed and approved by our university’s Compliance Department. The process involves setting up a server with a random hostname specifically for geocoding patient addresses. When an API call is made to an external geocoding service, the service may store patient addresses and referrer hostnames for auditing purposes, posing additional risk of patient reidentification. Utilizing a random hostname anonymizes the call such that our organization cannot be identified and linked to the patient addresses sent. This process takes an average of 0.27 s per address to geocode a sample set of addresses. Once this new process is fully implemented, we will geocode and geo-enrich our EHR data once a week.

Ethical issues remain inherent with the use of patient geographical data, and geolocation data are an element of PHI when linked to patients or HPOs [13]. We recommend becoming familiar with decisions associated with the geocoding process [14], variability of positional accuracy, geocoding methods [15]. the use of different geographical units when matching address [16], as well as published practices and protocols for internet geolocation [9,10,17] Institutional interpretation of HIPAA and privacy policies varies, and patient geolocation approaches should be evaluated by appropriate officials prior to implementation [9].

Employing secure strategies to geocoding EHR data allows for the benefits of geo-enrichment of patient data while minimizing privacy and security risks. Once securely geocoded, data can be safely enriched with any place-based measures to study the impacts of SEDoH and design and prescribe interventions to yield better health outcomes. We have outlined a framework for secure, compliant geo-enrichment of patient data that can be adapted and implemented at other institutions. Increasing consideration of SEDoH in both research and clinical practice can ultimately reduce health inequities and improve outcomes for marginalized populations.
